# Commentary: Greater addition of neurons to the olfactory bulb than to the cerebral cortex of eulipotyphlans but not rodents, afrotherians or primates

**DOI:** 10.3389/fnana.2015.00084

**Published:** 2015-06-23

**Authors:** Romain Willemet

**Affiliations:** IndependentToulouse, France

**Keywords:** adjustment effect, olfactory bulb, brain evolution, mammals, microsmia

Mammalian brains differ in many dimensions and how to compare them is one of the fundamental questions of comparative neuroscience. In a recent paper related to this issue, Ribeiro et al. (2014) examined the number of neurons within the olfactory bulb in several species of mammals. Among other things, Ribeiro and collaborators estimated that the olfactory bulb in humans contains as many neurons as the olfactory bulb of the largest species of Eulipotyphla. Assuming that “total numbers of neurons are a valid proxy for total information processing capacity in each structure” the authors concluded that “given the large absolute number of neurons predicted to compose their olfactory bulb, compared to macrosmatic eulipotyphlans, humans (and other primates) should no longer be considered microsmatic.”

It is first important to note that the concept of micro/macro-smia is problematic. Sensitivity, acuity, selectivity, and the number of odorants that can be processed simultaneously are certainly major dimensions of olfactory ability that cannot be accounted for by an unidimensional, discrete factor. Moreover, the concept is relative to other mammalian groups and thus does not represent an absolute index of a species' olfactory ability. Finally, many factors other than the size of the olfactory bulb determine an animal's capacity to smell (such as the structure of the respiratory/olfactory system, Smith et al., 2004). These fundamental issues aside, the question here is whether the fact that our olfactory bulb contains as many neurons as those of species known for their olfactory abilities can be taken as evidence that humans and other primates also have large olfactory abilities.

The short answer to this question is probably no. There are taxon specific differences in the organization of most brain structures that makes comparisons based on a single variable problematic (Willemet, 2012). For example, primates olfactory bulbs seem to have a larger number of glomeruli for each functional olfactory receptor genes compared to rodents (Maresh et al., 2008; Moriya-Ito et al., 2015).

A more specific way to address this question, which is the main point of this paper, is to consider one possible consequence of the fact that brain structures are connected to each other within the brain network. The logic is that, when some brain structures increase their size due to adaptive process, it might be necessary for the other structures to increase their size (more precisely, their number of cells and connections) in order to maintain their relative influence in the brain process. Such a structure increases its size not because its functions are needed in the new species more than they were in the ancestor, but because the increasing number of axons and synapses in the whole brain as well as the increasing distance between brain regions would “dilute” too much its influence in the brain network if it was to keep its original size. In its most neutral form, this “adjustment effect” could possibly take the form of an increased number of redundant neurons and connections that would let the processing capacity of a structure relatively unchanged.

Returning to Ribeiro et al.'s study, this hypothesis suggests that the number of neurons in the human olfactory bulb does not necessarily represent the potential for olfactory abilities because a large part of these neurons may be there to keep the influence of the olfactory bulbs in the human brain, rather than for supporting increased olfactory ability. The relatively large number of glomeruli in anthropoids' olfactory bulbs (Maresh et al., 2008; Moriya-Ito et al., 2015), despite primates having a relatively low number of functional olfactory receptor genes (Niimura and Nei, 2007), might be the kind of redundancy predicted by the adjustment hypothesis.

Importantly, this hypothesis does not predict that the olfactory abilities of primates including humans are poor, or even poorer than those of Eulipotyphlan. Instead, it suggests the possibility that since the first primates (or anthropoids), there may have been only limited, or even inexistent direct selection on olfactory abilities, even though the olfactory bulb generally became bigger. In other words, there may be no correlation between the number of neurons in the olfactory bulb and olfactory ability in primates. This hypothesis is fully compatible with a recent framework on brain evolution which, in stark contrast to the traditional approach, explains species variation in brain structure size as mainly resulting from adaptive pressures directed toward individual structures (Willemet, 2013). Unfortunately, there is no comparative study on the four dimensions of olfactory abilities noted above, which makes it impossible to determine, which, between direct adaptation and indirect adjustment; most affected the size of the olfactory bulb in primates. However, the few and limited comparative studies to date do not present any clear evidence of an effect of the size of the olfactory bulb on olfactory sensitivity in primates (e.g., Laska et al., 2007), which tends to support the adjustment hypothesis.

If the present hypothesis is valid and applies to the olfactory bulb (its effect should depend on the connectivity of each structure), there should be anatomical and functional differences between primate and eulipotyphlan olfactory bulbs with similar number of neurons (in addition to the size differences revealed by Ribeiro et al., 2014). In this regard, it is interesting to note that the number of neurons in the human olfactory bulb is about half that predicted by Ribeiro et al. (Oliveira-Pinto et al., 2014). Unfortunately, the small number of species in Ribeiro et al.'s study does not allow to determine whether this represents a departure from the primate trend (and see Willemet, 2012 for arguments against comparative analyses at the order level in primates).

In any event, in addition to the fact that the small relative size of the olfactory bulb is not proof than humans and other primates are microsmatics (Willemet, 2013), the fact that primates olfactory bulbs have as many neurons as the olfactory bulbs of some macrosmatic species is not necessarily proof that both groups have comparable olfactory abilities. More generally, the adjustment effect might be a fundamental factor for understanding the evolution of brain structure size in birds and mammals (Willemet, in prep).

## Conflict of interest statement

The author declares that the research was conducted in the absence of any commercial or financial relationships that could be construed as a potential conflict of interest.
